# Bacterial Pigments and Their Multifaceted Roles in Contemporary Biotechnology and Pharmacological Applications

**DOI:** 10.3390/microorganisms11030614

**Published:** 2023-02-28

**Authors:** Himani Agarwal, Sneh Bajpai, Arti Mishra, Isha Kohli, Ajit Varma, Mireille Fouillaud, Laurent Dufossé, Naveen Chandra Joshi

**Affiliations:** 1 Amity Institute of Microbial Technology, Amity University, Noida 201313, India; 2 Department of Botany, Hansraj College, University of Delhi, Delhi 110021, India; 3 Chemistry and Biotechnology of Natural Products, CHEMBIOPRO, Université de La Réunion, ESIROI Agroalimentaire, 15 Avenue René Cassin, CS 92003, CEDEX 9, F-97744 Saint-Denis, France

**Keywords:** biocolorant, biotechnology, pigments, pharmaceutical applications

## Abstract

Synthetic dyes and colourants have been the mainstay of the pigment industry for decades. Researchers are eager to find a more environment friendly and non-toxic substitute because these synthetic dyes have a negative impact on the environment and people’s health. Microbial pigments might be an alternative to synthetic pigments. Microbial pigments are categorized as secondary metabolites and are mainly produced due to impaired metabolism under stressful conditions. These pigments have vibrant shades and possess nutritional and therapeutic properties compared to synthetic pigment. Microbial pigments are now widely used within the pharmaceuticals, food, paints, and textile industries. The pharmaceutical industries currently use bacterial pigments as a medicine alternative for cancer and many other bacterial infections. Their growing popularity is a result of their low cost, biodegradable, non-carcinogenic, and environmentally beneficial attributes. This audit article has made an effort to take an in-depth look into the existing uses of bacterial pigments in the food and pharmaceutical industries and project their potential future applications.

## 1. Introduction

Natural dyes have gained more concern for their application because they are more compatible with the environment due to their better biodegradability. Natural dyes have been utilised for a very long time; they served as colouring agents in the earliest known human civilizations. However, as synthetic colours were introduced to the market, they started to lose their charm. These synthetic dyes were much cheaper, user-friendly, came in a variety of colours, and were reproducible compared to their natural alternatives. The plant-based dye industries collapsed at the beginning of the twentieth century, whereas the synthetic dye business flourished as a result of recent advances in the field. The industries using synthetic dyes ended up dumping the toxic chemical wastes into freshwater sources, such as rivers and ponds, which led to algal blooms, ultimately contributing to greenhouse gases [1]. These industrial pollutants were hazardous and carcinogenic for consumers as well. It was not until recently that the synthetic dyes ill effects started to surface, and industries were forced to look in a new direction. Because of these demerits of synthetic dyes, their application nowadays is considered “toxic contaminants” and are, thus, less oftenly used [2]. Natural dyes made from plants are already in the market, but there are disadvantages that must be taken into consideration. It might be expensive to grow plants specifically for dyeing purpose. Furthermore, plant-based pigments are rapidly denatured in the presence of changed pH and might not provide batch reprodubility. Consequently, researchers used a different strategy involving microbes [3].

Bacteria, yeasts, fungi, and algae are a few of the microbes that typically generate natural colours. Several categories that have been documented to be synthesised by these microorganisms, including carotenoids, flavins, phenazines, violaceins, melanins, and many others that are mentioned in the paper later. These pigments are essential for the microorganisms’ innate ability to adapt to harsh environmental circumstances and perform specific cellular functions (for example, photosynthesis in photosynthetic micro-organisms) [4]. Such naturally synthesized pigments are now used in various industries, such as textile, cosmetics, pharmaceuticals, and food, because of their exotic properties and advantageous characteristics, which are useful for both human health and the environment [5].

The global market for microbial pigments has been assessed for a number of pigments; for instance, the estimated market for carotenoids was $1.7 billion USD for the year 2020 and is likely to expand at a pace of 2.6 percent with an expected range of almost $2 billion USD in the coming years. According to AMR (2021) reports, astaxanthin, a kind of carotenoid, had a market size of $192.5 million USD in the 2020 and will experience a exponential increase to about $228.4 million USD by the year 2027 [6,7]. Currently, 80–90% of the carotenoid synthesis is performed by chemical synthesis, since it is of lower cost ($250–2000 kg^−1^) when compared to plant-derived carotenoids ($350–7500 kg^−1^) [8]. On the other hand, the carotenoid-containing biomass from many different microorganisms, such as, for example, algae (*Haematococcus* sp., *Chlorella* sp., etc.), are sold at $40–50 USD per kg in the open market [9]. Thus, microbe-derived pigments can take over the global market and cut off synthetic and plant-derived pigments in the present scenario. This review discusses a detailed overview of bacterial pigments, their extraction and purification process, and their application in food, cosmetics, and biomedical sciences.

## 2. Overview of Natural Pigments, Plant Pigments and Microbial Pigments

**Figure 1:**
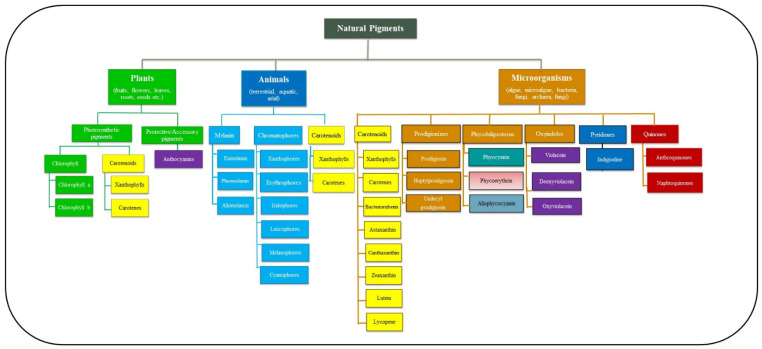
Classification of natural pigments on the basis of organisms. The natural pigments can be obtained from different organisms involving plants (photosynthetic and protective pigments), animals (Chromatophores and carotenoids), and micro-organisms (prodigines, phycobiliproteins, oxyindoles), which have various pharmaceutical and industrial applications.

A pigment can be defined as a chemical compound capable of absorbing a fraction of photons falling on it and transmitting the rest, which falls in the detection range of human eyes [10]. However, these compounds are not the same as phosphorescent, fluorescent and other luminescent materials, with inherent, light-emitting properties. Pigments selectively absorb a particular wavelength, which imparts them with their distinctive colours, and since they possess this property, they are used to colour plastics, fabrics, cosmetics, foods, and ink/paints. The pigment is any coloured material produced by a living organism (Figure 1). Physical organs, such as nails, hair, skin, iris, and fur, also bear unique pigments known as melanin [11]. Additionally, other organisms, such as reptiles (involving chameleon, garden lizards), amphibians (such as frogs), teleost (involving fishes like kill fish, eel, catfish, minnow), and insects (involving stick insect, grasshoppers, lake flies), have specialized pigment-producing cells called chromatophores that enable them to change the colour of their skin under specific conditions. Thus, pigmentation in biological systems aids the survival of organisms by enabling them to mimic and camouflage with their immediate environment [12].

In some cases, pigments might also serve as the basis for sexual selection, signalling, and aposematism [13]. For example, in guppies, the orange spots are determined by carotenoid uptake in their diet. Males with a low-carotenoid diet have dull orange spots and are less preferred by females over the males with high-carotenoid uptake [14]. Pigments (chlorophyll) aid in complex chemical cascades, such as photosynthesis in plants. Pigment colours should not be confused with structural colours; structural colours are iridescent, whereas pigment colour remains the same from different angles. 

Prokaryotes are known to synthesize pigments, just like animals and plants do. Some of these pigments aid in the synthesis of complex carbohydrates by photosynthetic bacteria, while others protect them from ultraviolet (UV) damage [15]. Prokaryotes can be divided into two major categories: autotrophic prokaryotes and heterotrophic prokaryotes. Autotrophic prokaryotes bear pigments that help them participate in photosynthesis; chlorophyll, carotene, and xanthenes are the most commonly found pigments in autotrophic prokaryotes. The heterotrophic prokaryotes produce some accessory pigments, which helps in the survival of these organisms in extreme niches. For example, the membrane bound, yellow-coloured pigment xanthomodin is secreted naturally by the bacteria *Xanthomonas oryzae* pv. *oryzae*. In addition, xanthomodin has been shown to possess photodamage protection properties to the bacteria [16]. The biosynthesis of these accessory pigments is crucial for the taxonomic characterization and identification of novel bacteria and helps establish genetic relatedness amongst new species and the pre-existing ones. The major types of bacterial pigments with different therapeutic uses are shown in Table 1.

## 3. Ecological Distribution of Microbial Pigments

Pigmented micro-organisms, also known as chromogenic microorganism, belonging to, e.g., bacteria, microalgae, archaea (mainly haloarchaea), have been isolated from diverse environmental and geographical conditions, including terrestrial, aerial, and marine locations [32]. Research is being conducted extensively on coloured microorganisms isolated from marine habitats, including seawater, marine sediment, sponge [33], sea ice (including Algoriphagus), sun saltern, microbial mats [34] and many other locations due to the variety of colours and novelty. Several pigment-producing microorganisms have also been found in a variety of stress environments, such as lava caves [35], hot springs [36], etc., in addition to normal environments. There are also specific geographic locations where the frequency of the pigmented microorganisms is higher than other occurrences. For example, Hermansson et al. (1987), discovered that the frequency and occurrence of pigmented bacteria were higher at the air–water interface than bulk water [37]. Additionally, glaciers, ice cores, salt lakes, and deep-sea hydrothermal vents are a few locations with large concentrations of coloured microorganisms [38]. Thus, it can be shown that the pigment-producing microorganisms are extensively dispersed around the world, providing enormous opportunity for scientists to learn about their medicinal and industrial uses.

## 4. Advantages of Microbial Pigments over Synthetic Pigments

Applications of bacterial pigments are more favoured than synthetic pigments because of a number of advantages. Bacterial pigments are simple to grow and secure for use by humans. Furthermore, their extraction methods and scaling up processes are more economical. Additionally, pigments are secondary metabolites created by a living creature that support the cell in numerous ways, including photosynthesis, UV protection, defence against competing species, and even energy-storing molecules [39]. They are a good contender for the current pigment industry because of their ease of growing, resistance to temperature and pH changes, variety, and non-toxic/eco-friendliness. Additionally, bacterial pigments possess great medicinal qualities, making them even more deserving of replacement for synthetic dyes. For instance, bacterial pigments, such as zeaxanthin, astaxanthin, yellow and orange carotenoids, prodigiosin, violacein, pyocyanin, and actinorhodin are the subject of intense research for their possible use in modern medicine [39]. The ability to biosynthesize any natural pigment using a bacterial host cell is another advancement in recombinant DNA technology that has further cemented bacteria’s place in the pigment industry. Now, rather than growing quintals of plants for their pigments, bacterial culture can achieve the same results [9]. This simplistic beauty of working with prokaryotes attracts more research into the topic.

## 5. Pigment Production from Bacteria and Genes Involved in Pigment Production

**Figure 2:**
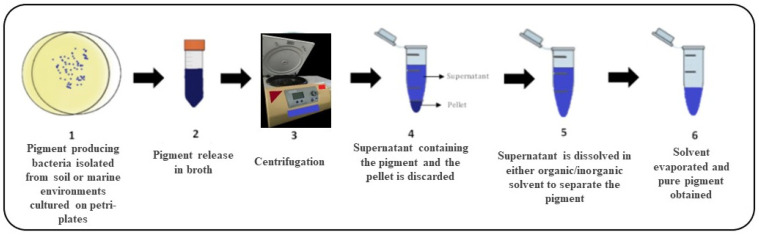
Extraction and purification of microbial pigments. The process of production, extraction, and purification of microbial pigments is explained in the figure and is numbered serially from 1 to 6 respectively.

Bacterial pigments fall into one of two categories: soluble pigments that quickly permeate into the surrounding medium and are referred to as extracellular pigments; and insoluble pigments are confined to the interior of the cell and are referred to as intracellular pigments. In extracellular pigments, the liquid culture can be directly processed through chromatography to isolate the pigment. On the other hand, intracellular pigments first require the culture to be sonicated; this breaks the cell open to release the pigment into the surrounding media (Figure 2).

Bacterial pigments are secondary metabolites synthesized only when the bacterial species are grown in appropriate growth conditions. The pigments, such as orange carotenoids, protect the cell from the damage caused by harmful ultraviolet light when the bacterial culture is exposed to direct sunlight [19]. Some pigments are made in the lag phase where the cell is experiencing resource exhaustion and thus, pigmentation is also sensitive to the kind of medium utilized for production. Different bacterial species have special growth requirements under which they produce specific pigments. The percentage yield can be calculated on a laboratory scale by closely monitoring the purified pigment’s absorbance; the more the absorbance, the better the pigment production efficiency will be. The maximum yield and quality of the pigments can be simply attained via media optimization and standardization of incubation conditions [40,41]. Bacterial culture will be unable to surpass the lag until the right conditions are provided; otherwise, the quality and quantity of the pigment produced will be of an inferior kind.

Molecular cloning can enhance pigment production by developing high yielding strains that will reduce the cost of maintaining stringent fermentation conditions and produce more pigment per unit mass. However, the type of pigment being purified depends greatly on the downstream purification of the pigment from the fermentation media. Therefore, a proper purification scheme is required for pigment purification. Since most bacterial pigments are insoluble in water, they are selectively solubilized in organic solvents, such as methanol, ethanol, acetone, ethyl acetate, or hexane. Once the pigment is obtained in the organic solvent, the organic solvent is allowed to evaporate. The powdered residue left behind is our pure pigment and can be used for functional analysis [42]. There are different types of pigments, such as canthaxanthin, β-carotene, violacein, phenazines, etc. Each pigment is produced by different kinds of micro-organisms via a tightly regulated biochemical pathway catalysed by a number of enzymes that are encoded by other genes, respectively. A list of various pigments and the genes involved in their synthesis is given in Table 2, respectively.

## 6. The Utilization of Genome Engineering Techniques to Enhance Bacterial Pigment Production

Biosynthesis of several industrially important pigments, such as carotenoids, lutein, zeaxanthin, etc., can be hosted in microorganisms using modern genetic engineering techniques that do not naturally produce them, or if they do, they are made in minute quantities [9]. Therefore, various strategies have been postulated to develop genetically engineered microorganisms to synthesize the desired pigments, such as over-expression of the key enzymes involved in the pigment biosynthesis, insertion/deletion of specific genes, etc. Such genetic modifications not only help in strain improvement, but also help in mitigating the toxic issues, which are caused by synthetic dyes.

*Corynebacterium glutamicum* has been metabolically engineered for the co-production of a secreted amino acid (L-lysine) along with a cell-bound carotenoid (β-carotene) using two feedstocks, namely, xylose and arabinose. Furthermore, a genetically engineered strain of *C. glutamicum* for the transcriptional repressor gene *crtR* has also been developed to overproduce the carotenoid decaprenoxanthin [50]. In addition, *Escherichia coli* has been genetically engineered to obtain several pigments, such as β-carotene, zeaxanthin, etc. For example, via modification of a single gene of ATP synthesis, pentose phosphate, and TCA cycle, a modified *E. coli* has been constructed to synthesize β-carotene [39].

Recent advances in genetic engineering have helped scientists engineer certain microorganisms, such as *E.coli* and yeast, for large-scale carotenoid production. For example, an engineered *E.coli* has been constructed to synthesize zeaxanthin from lycopene using two fusion protein-mediated substrate channels and introduce tunable intergenic regions to express *crtY*, and *crtZ* genes encode for the enzyme lycopene β-cyclase and a β-carotene hydroxylase, respectively [51]. *Rhodobacter sphaeroides* was genetically engineered for the production of lycopene by many modifications; firstly, its *crtI_3_* gene was replaced by *crtI_4_* gene from *Rhodospirillum rubrum*. Secondly, the *crtC* gene was deleted and, finally, the *zwf* gene was knocked out along with the integration of *dxs* gene, which blocked the competitive pentose phosphate pathway and reinforced the methyl erythritol phosphate pathway [52]. Thus, genetically engineered micro-organisms can be extensively employed for the large-scale production of pigments with many therapeutic and industrial uses. The different types of genetically engineered bacteria for the industrial production of pigments are given in Table 3.

## 7. Application of the Bacterial Pigments in the Pharmaceutical Industry

Since the discovery of antibiotics, the fatality due to bacterial infections has drastically reduced. Over the past decades, not only has the average lifespan of human beings increased, but we have also learned how to tackle health care emergencies. Thanks to our ever-expanding pharma industry, which invest in the research and development of new drugs and manage their production and circulation amongst the human population. However, as years pass by, our knowledge of modern medicine still stands incompetent in front of the present-day challenges. Due to the injudicious circulation of heavy antibiotics, multidrug-resistant (MDR) strains of pathogenic microorganisms have developed. This is one of the challenges that have rendered us helpless. Another one is the unsolved puzzles of diseases, such as cancer, which lay an irreversible impact on patients and their families. To tackle the serious issues of antimicrobial resistance and expensive healthcare, the pharma industry was compelled to look into a new direction—‘bacterial pigments’. Bacterial pigments in recent years of research have revalued their importance; they might be the next big breakthrough in the field of modern medicine. The following sections discuss a brief account of recent developments in the pharma industry using bacterial pigments for therapeutics purposes.

### 7.1. Anti-Tumour Effect of Bacterial Pigments

A hallmark of cancerous tissues is their unceasing cell division, which causes cells to start to accumulate in a small area and eventually form tumours. Carcinogenic pollutants, unhealthy food habits, oncogenic bacteria and viruses, as well as several more unidentified causes, are only a few of the factors that cause malignant tendencies in somatic cells [56]. In recent years, cancer cases have increased exponentially; it is the second leading cause of death worldwide. The reason behind it is the uncontrolled exposure of individuals to industry generated carcinogenic compounds [57]. Chemotherapy is available for the treatment of solid tumours. Still, these drugs are not selectively toxic to cancerous cells since the cancer cells do not differ from healthy cells morphologically. Therefore, along with killing the tumour cells, some of the neighbouring healthy cells are also damaged. This drawback of present-day chemotherapy fuels the search for a new anti-tumour drug.

**Figure 3:**
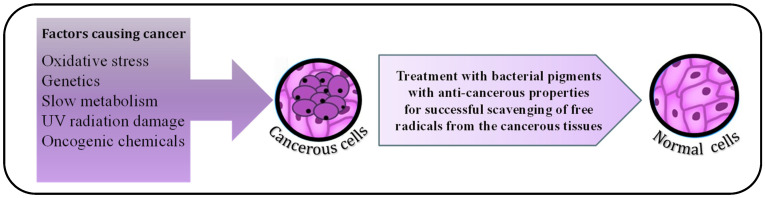
Antioxidant and anti-cancer activity of bacterial pigments. There are various causative reasons leading to cancer involving genetic mutations and various mutagens, such as UV rays and chemicals. Bacterial pigments have found their use as substitutes for cancer treatment, as they have anti-cancer properties, such as scavenging of free radicals from the cancerous cells.

Many bacterial pigments have demonstrated a significant anti-tumour activity; for instance, *Deinococcus radiodurans*, a radioresistant bacteria, produce red, non-photosynthetic pigment, deinoxanthin, which is structurally a carotene. Normal cells, during their functioning, generate free radicals, which upon accumulation in the cell, can cause cancer [58]. Deinoxanthin is a stronger reactive oxygen species (ROS) scavenger than carotenes, such as lycopene, β-carotene, and xanthophylls, such as zeaxanthin and lutein [22]. Recent research demonstrated that deinoxanthin is effective in inducing apoptosis in HepG2 (human liver cancer cell line), PC-3 (prostate cancer cell line), and HT-29 (human colon cancer cell line) (Figure 3). Marine microbiota is under extensive investigation since many pigments isolated from marine Actinomycetes have also shown cytotoxic activity against multiple human cancer cell lines. Actinomycetes, such as *Saccharomonospora azurea* [59] and *Streptomyces* spp. [60], such as *Streptomyces hygroscopicus* subsp. *Ossamyceticus* [61] and *Streptomyces torulosus* [62], are known to produce blue, green, yellow, orange, violet, brown, and red pigments, out of which the red coloured pigment is of the most therapeutic value [63]. The red pigment belongs to the family of prodigiosins, which is synthesized by *Streptomyces* and *Serratia marcescens.* Prodigiosins induce mitochondria-mediated cellular apoptosis and caspase activation, and they can even arrest the cell cycle in the late G1 phase.

Numerous species of the genus *Streptomyces* are known to produce many bioactive compounds and pigments that have demonstrated anti-tumour activity. In the search for a novel anticancer medication, *S. aburaviensis* and *S. psammoticus* are preferred possibilities [64]. *S. gresioaurianticus* JUACT 01 produces a yellow-coloured pigment that induces apoptosis in HeLa and HepG2 cell lines and is nontoxic to the human lymphocytes and is also a promising candidate for further investigations [65]. Violacein is a naturally occurring bis-indole pigment with anti-tumour activity and is isolated from *Chromobacterium violaceum.* Violacein induces apoptosis in cancerous cell lines by activating caspase 8 and transcription of nuclear factor kappa B (NF-Κb), target genes/p38 mitogen-activated protein kinase; this mechanism resembles the TNF-α activation cascade [28]. The regulated cytotoxicity of natural pigments makes them ideal for cancer therapy. Another rich source of anti-tumour bioactive agents is halo-archaeal carotenoids produced by the haloalkaliphilic archaeon *Natrialba* sp. M6. The in vitro studies demonstrate effective anticancer activity via apoptosis-dependent and blockage matrix metalloprotease (MMP)-9, responsible for cancerous angiogenesis and metastasis [66]. Pyocyanin, a phenazine actively produced by *Pseudomonas aeruginosa*, was initially observed to have cytotoxic effects on human pancreatic cancer cell lines (Panc-1) [67]. Similar anti-cancer property was exhibited by pyocyanin on glioma cells of 66.34%, human hepatoma cells (HepG2). Pyocyanin primarily prevents the growth of cancer cell lines by producing large amounts of ROS, such as hydrogen peroxide (H_2_O_2_) and superoxide (O_2_^–^), which cause significant oxidative stress and cell damage [68].

### 7.2. Antimicrobial Action of Bacterial Pigments

Bacterial infections are a constant threat to the human community. Due to their fast propagation and ability to mutate rapidly, bacteria often gain resistance against antimicrobial drugs. Additionally, this antimicrobial drug resistance is a bane to public health, as controlling these infections is not only difficult but monetarily straining. Hence, researchers are now switching to more novel antimicrobials, such as bacterial pigments to combat antimicrobial drug resistance. For instance, a deep red pigment produced by *Streptomyces* sp. JS520, is shown to provide adequate protection against bacterial infection caused by *Bacillus* and *Micrococcus* [69].

Furthermore, spectrometric analysis of the pigment reveals high concentrations of undecylprodigiosin (an intermediate compound in the biosynthesis of prodigiosin), inhibiting biofilm formation in pathogenic bacteria, thereby preventing infection [24]. Deep-sea sediment strain *Streptomyces* sp. SCSIO 11594 is also known to produce significant amounts of undecylprodigiosin, which demonstrated effective antimicrobial activity against methicillin-resistant *Staphylococcus epidermidis* shhs-E1 [25]. In addition, undecylprodigiosin is effective against *Proteus mirabilis*, *Salmonella* sp., *Shigella* sp. and *Enterococcus* sp. but is not effective against *Escherichia coli* and *Klebsiella pneumonia* [60]. With all these shreds of evidence in hand, more research can be put into preparing undecylprodigiosin as a novel candidate for antimicrobial drugs.

*Streptomyces* genus synthesizes many other antibacterial pigments, such as actinorhodin [70] and tetracycline [71]. Out of these, tetracycline is already a FDA-approved drug, and a bactericidal molecule hinders the pathogen’s protein synthesis. In nature, many compounds are present whose production is based on quorum sensing; that is, the organism produces these compounds for the selective proliferation of its kind and usually has an inhibitory effect over the rival species [72]. In addition, a green-coloured pigment produced by *Bacillus cereus* strain called cerein; and it is bactericidal for other *Bacillus cereus* strains [73]. Recent investigations have been conducted to study the antibacterial potential of halophilic bacterial pigments. Being natural products, these pigments have no side effects on the consumer, making them more desirable for large scale antibiotic production. Pyocyanin also possesses anti-bacterial activities against several Gram-positive and Gram-negative bacteria. This property is due to the interaction of pyocyanin with the respiratory chain of cell membrane, and thus the bacterial cells lose their ability to perform their metabolic transport process actively [74]. Pyocyanin had an inhibitory effect on gram-positive bacteria like *Staphylococcus aureus*, *Staphylococcus saprophyticus*, and *Enterococcus faecalis* even at lower concentrations, whereas for gram negative bacteria, for example *Klebsiella pneumonia*, *Proteus mirabilis* and *Morganella morganii* there was no inhibitory effect even at higher concentrations [27,75].

### 7.3. Antiviral Activity of Bacterial Pigments

Viral infections are one of the most challenging and trickiest infectious diseases to treat. The virions are inactive until they find a suitable host for their proliferation; thus, it is tough to control or limit the virion when it has not infected the host already. Once the virus infects the cell, it either follows the lytic cycle or the lysogenic cycle, which involves the synchronized participation of many enzymes and receptors [76]. Most antiviral drugs target these surface receptors and enzymes that allow the virus to enter the host cell and take over its DNA replication machinery. The major limitation of this approach is that viral surface receptors are homologous to the host cell surface receptors, resulting in inaccurate drug targeting. Such medications lead to some significant side effects for the patient. For instance, antiretroviral therapy (ART), which is given to cure HIV infection, has been reported to cause specific side effects, such as hypersensitivity, anaemia, weight loss, fatigue, headaches, peripheral neuropathy, etc. Additionally, the ever-changing genetic makeup of viruses also presented a challenge of antiviral drug resistance [77]. Thus, research investigations have been steered towards a direction of safer and more effective antiviral alternatives available in nature.

Tri-pyrrole produced in bacteria, prodigiosin, demonstrates antiviral activity when tested against cells infected with *Bombyx mori* nucleopolyhedrovirus (BmNPV) (a viral infection causing significant economic losses to the silk industry); it stops the early transcription of viral genes and selectively kills the virus infected cells. In addition, it prevents viral DNA synthesis and inactivates the virus-mediated fusion proteins [78]. *Serratia marcescens* is also known to produce a different class of prodigiosins, which have shown effective antiviral activity against herpes simplex virus (HSV). The so-far understood mechanism behind the antiviral activity is due to the accelerated cell death via the inhibition of Akt and NF-Kb signalling pathway, i.e., the viricidal compound degrades the host signalling pathway essential for the survival of the herpes virus in the host cell [79].

The pigments, such as violacein produced by *Chromobacterium violaceum*, show antiviral activity against HSV-1, poliovirus, and simian rotavirus SA11 by slowing down the viral replication efficiency [29,80]. The violacein extracted from *Janthinobacterium lividum* XT1 has also shown anti-viral properties [30]. All the bioactive compounds mentioned above are strong candidates, ready for further research to invade the untapped market of pigment-based antivirals.

### 7.4. Antifungal Activity of Bacterial Pigments

Fungal infections are the most common among immunocompromised, diabetic and immunodeficiency virus-infected populations [81]. Unlike bacterial and viral pathogenesis, fungal pathogenesis is not complex, and fungal infections are comparatively easier to control. However, as the diseases prevalence has increased, more and more research has been performed to create potent new drugs to treat fungus infections. The main complexity in studying antifungal agents is that the drug should not interfere with the host’s cellular mechanisms. Both the host and the fungal pathogen, being eukaryotes, make it difficult to trace and to target unique biomolecules, which will selectively harm the pathogen and not the host [82].

Bacterial symbionts are known to contribute to amphibian skin defence. The analysis of the cutaneous microflora of frogs revealed the presence of two pigment-producing bacteria, *Janthinobacterium lividum* (producer of violacein) [31] and *Serratia plymuthica* or *S. marcescens* (producer of prodigiosin) [83]. The pigment isolated from these bacteria shows effective antifungal activity against the cultures of chytrid fungi *B.salamandrivorans* (*Bsal*) and *Batrachochytrium dendrobatidis* (*Bd*), with violacein being more significantly able to inhibit the fungal cultures at MIC (minimum inhibitory concentration) of 15 μM. *Janthinobacterium lividum* also produces other compounds, such as indole-3-carboxaldehyde, which is colourless as such, but, under certain physiological conditions, it turns purple and also demonstrates antifungal activity [31]. A yellow-coloured pigment produced by *Bacillus gibsonii*, isolated from the lichen *Dirinaria aegialita*, shows selective antifungal activity against economically essential fungi, and, with further investigations and clinical trials, it can be used as a prescription antifungal drug. It can also be formulated as an antifungal ointment [84].

Similarly, *Neisseria* spp. create a distinctive red pigment that has excellent antifungal activity against a few key fungal species that are harmful to humans, including *Aspergillus* spp., *Candida* spp., and *Trichoderma* spp. [85]. Furthermore, *Pseudomonas aeruginosa* cultures produce extracellular pyocyanin, exhibiting potential antifungal activity without harming human consumption. The concentration of mycotoxins produced by *Fusarium graminearum* was seen to decrease significantly by pyocyanin produced by *P. aeruginosa*, thereby inhibiting the growth of the respective fungus [86]. Pyocyanin shows antifungal activity against *Candida albicans*, *Candida tropicalis*, *Candida glabarata*, *Cryptococcus neoformans, Aspergillus fumigatus*, and *Fusarium oxysporum* through the arrest of electron transport chain [87,88,89]. A range of psychrophiles also provides an ever-expanding potential of being employed as non-toxic antimicrobial producers. For example, the isolates of *Rhodonellum psychrophilum* GL8 collected from Pangong Tso, Leh Ladakh, India, produces a red pigment containing a mixture of 2-methyl-3-hexyl-prodigine, 2-methyl-3-butyl-prodigine, prodigiosin, anhydrorhodovibrin, 1-tetradecanoyl-2-hexadecanoyl-3-octadecanoyl, 4-didehydrorhodopsin, and alloxanthin compounds. These newly discovered red pigments showed antifungal activity against *S. aureus*, *C. albicans*, (MTCC 277, ATCC 90028), and *S. cerevisiae* (H1086). Furthermore, it demonstrated a synergistic effect as an antifungal agent when coupled with antifungal drugs, such as amphotericin B, vancomycin, luconazole, and erythromycin [90]. As concurred from the examples cited above, the natural bioactive pigments have huge potentials to be used as fungicidal drugs if more research and trials are directed towards this area of bacterio-pharmacology.

### 7.5. Antiparasitic Activity of Bacterial Pigments

Parasitic infections are one of the deadliest and fastest spreading vector-borne infections. These include diseases, such as malaria, leishmaniasis, encephalitis, helminthiasis, etc. These parasites infest the host’s body and deprive it of essential nutrients, thereby meddling with the host’s overall growth. Approximately one billion people, who are one-sixth of the total Earth’s population, have suffered from at least one form of parasitic infection [91]. These data themselves are worrying, but they do not stop here. Parasitic infections are not just limited to human hosts, but they extend their unwanted courtesy to animals and plants, thereby causing an economic depletion. Due to the complex pathogenesis and evolutionarily advanced virulence, it is always challenging to develop formulations harming parasites but are non-toxic to the host. An investigation conducted on psychrotolerant bacteria from the continent of Antarctica revealed that a few strains of *Pseudomonas* spp. produce bioactive pigments, which possess antiparasitic activity [92].

Studies such as these reflect the actual efficiency of bacterial pigments and reveal the untapped potential psychrophiles offer. Pigments such as violacein and deoxyviolacein are popular in the pharma industry as antimicrobials, but, in recent years, their antiparasitic activity (especially against *Plasmodium falciparum* strains and *Trypanosoma cruzi*) has gained attention. Efforts are being made to produce violacein on a large scale and exploit its use as an anti-plasmodium and anti-trypanosomal drug [93]. Many prodigiosin and their synthetic derivatives are also potent anti-protozoan compounds [31]. Additionally, bacterial pigments have shown promise in reducing plant nematode infestations. For instance, *Serratia marcescens* pigment inhibited the juvenile forms of *Radopholus similis* and *Meloidogyne javanica* at a low dose [94]. The evidence from these studies demonstrates the new opportunities bacterial pigments have to offer in combating the deadly parasites.

### 7.6. Bacterial Pigments as Immunosuppressors

After getting a solid organ transplant, the patient’s immune cells often treat the organ tissue as a non-self leads to organ rejection. Immunosuppressive drugs are given to such patients to lower their immune response, making them immune deficient in the processes [95]. A non-toxic bioactive pigment isolated from marine bacterium *Pseudoalteromonas denitrificans*, cycloprodogiosin hydrochloride, was found to stop the T-cell proliferation selectively. Other proapoptotic members of the prodigiosin are also believed to impact the murine splenocytes [96]. These were one of the first studies conducted, and it has opened the door to the possibilities of bioactive pigments to be used as immunosuppressive drugs. MS-02–063, a strain phylogenetically related to γ-proteobacterium *Hahella* sp., is known to produce a red pigment RP-063, which has also been reported to have immunosuppressive activity, and its immunosuppression activity is different from the rest of the well known compounds, and it inhibits lymphocyte proliferation and reduces the superoxide release from these cells [97]. Another prodigiosin, 2, 2′-[3-methoxy-1′amyl-5′-methyl-4-(1″-pyrryl)] dipyrryl-methene (MAMPDM), is known to inhibit B-cell and T-cell proliferation. MAMPDM induced apoptosis in Con A stimulated cells, demonstrating selective T-cell inhibition [98].

## 8. Application of Microbial Pigments in Food Industries

Vibrant and colourful food items appeal to the senses of people of all age groups. There are pieces of evidence that prove the early Egyptian and Roman empires used natural food colours in their food to make their product more appealing [99]. Food industries have also begun to rely on food colourants, and to keep costs down, they encourage the use of synthetically manufactured food colourants due to their stability and low price. However, synthetic food colours are made out of the by-products of petroleum wastes and are not health-benefiting. In some cases, the long term use of artificial colouring can ill impact the health of the consumers [100,101]. Plant-based colouring agents are often costly and unstable; hence, researchers are shifting their focus towards bacterial pigments since they are more stable and easier to produce than plant-based pigments [102]. However, when compared to synthetic pigments, microbial pigments exhibit reduced stability in specific environmental circumstances (light, pH, oxygen, UV, temperature), which causes them to reduce their shelf-life and lose their colour over time [102,103]. Thus, certain techniques have been developed, such as micro-encapsulation, preparations of nanoemulsion, or nanoformulations, which increase the market value of microbial pigments making them more durable for industrial applications [104,105,106]. With the knowledge of recombinant DNA technology, bacterial pigment production can be increased multifold. Microorganisms produce pigments, such as flavin, melanins, monascins, violaceins, carotenoids, quinies, and many others [32], which can be employed in the modern food industry, not only as colourants, but also as pro stabilizers due to their free radical scavenging activity [107]. For example, a pinkish-red pigment, astaxanthin, produced by the bacteria *Agrobacterium aurantiacum* and *Paracoccus carotinifaciens*, is an excellent antioxidant, which, when used as a colourant in food items, not only imparts an attractive colour, but also increases the shelf life of the product, thereby acting as a preservative as well [108]. Similar to astaxanthin is canthaxanthin, produced by *Bradyrhizobium* Sepp. This orange-coloured pigment is also a potent antioxidant currently being used in the food industry [109].

As discussed earlier, bacterial pigments also have many therapeutic properties associated with them; thus, the inclusion of such pigments in food products will enhance the visual appeal of the foods and fortify them with essential medicinal properties. For example, Heptyl prodigiosinis, a pink anti-plasmodial pigment (isolated from α-Proteobacteria), prodigiosin, a red anticancer pigment (isolated from *Serratia marcescenes*), and pyocyanin, a proinflammatory green pigment (isolated from *Pseudomonas* spp.) [27], are a few examples of the pigments already being used by the food industry, which are also having therapeutic applications. In addition, many pigments, such as staphyloxanthin (isolated from *Staphylococcus aureus*), trypanthirin (isolated from *Cytophaga*/*Flexibacteria* AM13,1 Strain) [110], and undecylprodigiosin (isolated from *Serratia marcescenes*), are under laboratory analysis and soon might be used in the food industry as a non-toxic, therapeutic food colourant. With more research investigations directed towards the search for new bacterial pigments and soon, a new class of immune-fortified foods might gain popularity, as these foods would be not only appealing, but also impart therapeutic immunity to the consumer, especially in the prevailing times where humanity is more than ever prone to infectious diseases and lifestyle disorders.

## 9. Application of Microbial Pigments in the Cosmetic Industry

Since synthetic dyes have toxicity and carcinogenicity issues, cosmetic industries also switch to safer alternatives. One of these is low cost yet highly efficient bacterial pigments. Most of these pigments are isolated from marine bacteria or extremophiles [111]. The degradation of the dermal and epidermal layer’s extracellular matrix is the primary factor behind the skin’s ageing, which is mainly controlled by the intrinsic factor (genetics and personal diet). However, the external environment (smoke, pollution, UV exposure, weather, etc.) contributes to the premature ageing of the skin. Amongst all the known pigments, β-carotenoids are the active ingredients used in anti-ageing creams. Carotenoid is a lipid-soluble pigment with a characteristic carrot-like orange colour produced by *Deinococcus radiodurans* [112], having an excellent capacity to prevent the production of ROS, which causes extensive damage to cells. Therefore, it is used in anti-ageing formulations, such as provitamin A. The anti-ageing property of cosmetics is due to high percentages of antioxidants. One of the pigments produced by *Haematococcus pluvialis* is astaxanthin, which is known to have excellent antioxidant properties. These antioxidants scavenge upon the free radicals produced by the cells [113]. The other two most commonly used antioxidants in the cosmetics industry are; myxol and saproxanthin. These pigments are members of the carotenoid family and are isolated from strains of marine bacteria belonging to the *Flavobacteriaceae* family [114].

Prolonged exposure to UV radiations causes dermatoheliosis, also known as photo-ageing [115]. In the long run, UV exposure can also cause DNA damage and can cause skin cancer. Hence, nowadays, moisturizersizers and sunscreens add pigments that reprimand the UV damage. Most of the extremophiles produce pigments that assist them in avoiding DNA damage. Scytonemin is one such pigment, a UV-A inducible pigment made by cyanobacteria that may aid in UV radiation protection due to its potential for absorption in the UV-A and UV-B range [116,117].

The modern-day cosmetic industry relies on bacterial pigments as effective preservatives since chemical preservatives can be harmful or toxic to the user and might decompose into undesirable compounds, rendering the product ineffective. A polyacetylene pigment, falcarindiol, obtained from the chloroform extract of *Crithmum maritimum* [118], has antimicrobial effects against bacteria *Micrococcus luteus* and *Bacillus cereus*, thereby imparting long shelf life to the product. However, bacterial pigments have still not been profoundly studied for their skin whitening, but astaxanthin, a carotenoid, is known to have depigmentation properties that aid in the lightening of the skin spots developed due to skin ageing by reducing melanin production [18]. Therefore, companies in cosmetic industries are investing more in researching marine bacterial pigments and utilization in preparing cosmetic concoctions, which are safe and more efficient in function.

## 10. Application of Microbial Pigments in Textile Industry

Since they are non-carcinogenic and safe for the environment, the textile industry now favours microbial pigments. Synthetic colours can have a harmful impact on your health in a number of ways, including skin responses and the emission of potentially dangerous compounds during synthesis. Prodigiosins, which are bright red in colour isolated from *Vibrio* sp., can be used for dyeing silk, wool, nylon and acrylics [119]. Additionally, prodigiosin, produced by *Serratia marcescens* SB08, has found its potential use as a natural dye for various fabrics, such as acrylics, silk, polyesters and cotton [120]. The fabrics dyed with such microbial-derived pigments also retained anti-microbial activities against microbes, such as *P. aeruginosa*, *E.coli*, and *Bacillus subtilis,* making the fabrics much safer for human use.

## 11. Structure and Bioactivity of Microbial Pigments

Sajjad et al. (2018) identified the structure–function relationship of prodigiosin and found that the function of microbial pigments is correlated with their structure and the bonds present in them [121]. They found that the strong antioxidant activity of prodigiosin is due to the pyrrole ring structure and conjugated double bond system. Montaner and Prez-Toms (2003) showed that the anticancer activity of prodigiosin is imparted by the C-6 methoxy moiety and A-pyrrole ring of the structure [122]. The antioxidant activity of violacein depends on its chemical structure, and it has two pyrrole rings conjugated with a benzene ring. It has been observed that the bond which is responsible for the violacein antioxidant is N7–H7 [123]. Violacein also has the capacity to absorb high-energy UV radiations (290–320 nm) due to its high resonance structure involving an aromatic compound conjugated with acryl groups. Violacein can absorb wavelengths in the range of 290–320 nm [124]. Carotenoids have properties such as antioxidant activity, light-absorption activity, and photoprotective action, which are conferred by their special polyene chain conjugated by a chromophore [125]. Thus, it can be concluded that the structure not only imparts the pigments their respective colors but are also responsible for their respective functions.

## 12. Conclusions and Future Perspectives

Bacterial pigments are secondary metabolites produced under stressful conditions, which harness many therapeutic properties. The bacterial pigments find their applications in the pharmaceuticals, food, and cosmetics industries. These pigments are effective against multiple mammalian cancerous cell lines and pathogenic microorganisms, thereby establishing their potential application in the pharma industries. These pigments also demonstrate antioxidant activity, thus acting as a potent free radical scavenger; these free radicals are crucial to be cleared from the cell for its healthy functioning. These pigments also find extensive use in the food industry as food colourants to increase their visual appeal. The pigments are also used as cosmetic additives due to their antioxidant properties; they make a significant constituent of anti-ageing creams. Bacterial pigments can secure a stable position in other commercial markets upon further research and development. Although bacterial pigments are eco-friendly and suitable for human consumption, they are not widely used in industrial circuits. This lack of awareness regarding bacterial pigments’ potential benefits is one of the biggest obstacles researchers haven’t yet overcome. Synthetic colouring comes in various shades and is thought to be cheaper to use; these two factors are sufficient for industries to disregard bacterial pigments and continue using artificial colourings. Research so far has already pointed out the therapeutic potential of bacterial pigments. With the right strategy applied, bacterial pigments can replace all of their synthetic counterparts used in the industries at the present moment. So far, we have been able to conquer just the tip of the iceberg, and there are a plethora of such pigment-producing bacteria, which have not yet been screened. Thus, our future objective should be to screen out the maximum number of pigments producing bacterial species from soil or marine niches. The more we screen for new species, the more the probability of finding unique pigments with enhanced therapeutic activities. This will reduce our dependence on antibiotics and other chemotherapeutic agents and reduce patients’ side effects due to their consumption. With the kind of lifestyle we are progressing into, bacterial pigments will protect us against diseases and reduce our dependency on synthetic and harmful colouring agents.

## Figures and Tables

**Table 1:** Bacterial pigments with various pharmaceutical applications.

S. No.	Pigment	Colour	Microorganisms	Clinical Uses	References
			1. Carotenoids		
(i)	Canthaxanthin	Orange to deep pink	*Bradyrhizobium* spp., *Lactobacillus pluvialis*	Antioxidant, Photoprotectant, Anti-cancer, Anti-inflammatory	[17]
(ii)	Astaxanthin	Red to orange	*Agrobacterium aurantiacum*, *Paracoccus carotinifaciens*	Antioxidant, Photo-protectant, Anti-cancer, Anti-inflammatory	[18]
(iii)	Zeaxanthin	Yellow	*Staphylococcus aureus*, *Flavobacterium* spp., *Paracoccus zeaxanthinifaciens*	Photoprotectant, Antioxidant	[19]
(iv)	β-Carotene	Red	*Rhodococcus maris*, *Rhodococcus ruber*	Anticancer, Antioxidant	[20]
(v)	Staphyloxanthin	Yellow	*Staphylococcus aureus*	Antioxidant	[21]
(vi)	Deinoxanthin	Red	*Deinococcus radiodurans*	Anti-cancer	[22]
			2. Prodiginines		
(i)	Prodigiosin	Red	*Serratia marcescens*	Anti-cancer, DNA cleavage, Immunosuppressant	[23]
(ii)	Undecylprodigiosin	Red	*Streptomyces* sp.	Antimalarial activity, Antibacterial, Antioxidant, Anti-cancer	[24,25]
(iii)	Heptyl Prodigiosin	Red	α-Proteobacteria	Antiplasmodial	[26]
			3. Phycobiliproteins		
(i)	Pycocyanin	Blue	*Pseudomonas* spp.	Cytotoxicity, Neutrophil apoptosis, Ciliary dysmotility, Proinflammatory	[27]
			4.Oxyindoles		
(i)	Violacein	Purple	*Chromobacterium violaceum*, *Collimonas* sp., *Duganella* sp., *Pseudoalteromonas* sp.	Antifungal, Antibacterial, Antiplasmodial, Anti-cancer	[28,29,30,31]

**Table 2:** The genes which are responsible for the biosynthesis of microbial pigments in different organism.

S. No.	Pigments	Genes Involved in the Biosynthesis of Pigments	References
1.	β-carotene	*crtE*, *crtY*, *crtI*, *crtB*	[43]
2.	Violacein	*vioA*, *vioB*, *vioC*, *vioD*, *vioE*	[44]
3.	Prodigiosin	*pig B*, *pigC*, *pigD*, *pigE*, *pigF*, *pigM*, *pigH*, *pigJ*	[23]
4.	Astaxanthin	*crtW*, *crtZ*	[45]
5.	Zeaxanthin	*crtE*, *crtB*, *crtI*, *crtY*, *crtZ*	[46]
6.	Staphyloxanthin	*crtO*, *crtP*, *crtQ*, *crtM*, *crtN*	[47]
7.	Canthaxanthin	*crtE*, *crtY*, *crtI*, *crtB*, *crtW*	[48]
8.	Pyocyanine	*phzE*, *phzD*, *phzF*, *phzB*, *phzG*	[49]

**Table 3:** Genetic engineering of micro-organisms for the production of important pigments.

S. No.	Microorganism	Type of Modification	Gene/Pathway Modified	Genes Origin	Pigment Produced	References
1.	*C. glutamicum*	Deletion	Sigma B factor (*sigB*)	-	Increase in Decaprenoxanthin β-carotene	[53]
		Over-expression	Sigma A factor (*sigA*)	-	Increase in Decaprenoxanthin Lycopene	
2.	*E. coli*	Pathway modification	MEP pathway	-	Lycopene	[43]
		Insertion of pathway	MVA pathway	-		
		Insertion	*mel* and ORF438 gene	*Streptomyces antibioticus*	Eumelanin	[54]
			*crtE*, *crtB*, and *crtI*	*Pantoea agglomerans*	Lycopene	
		Introduction of intergenic regions	*crtY* and *crtZ* genes	*Pantoea ananatis*	Zeaxanthin	[55]
		Deletion	*zwf* gene	*-*	Increase in β-carotene	[51]
			*ptsHIcrr* operon	*-*		
3.	*Rhodobacter sphaeroides*	Replace	*crtI_3_* with *crtI_4_*	*Rhodospirillum rubrum*	Lycopene	[52]
		Deletion	*crtC* gene			
		Knock out	*zwf* gene			

## Data Availability

Not applicable.

## References

[1] Kant R. (2012). Textile dyeing industry an environmental hazard. Nat. Sci..

[2] Tkaczyk A., Mitrowska K., Posyniak A. (2020). Synthetic organic dyes as contaminants of the aquatic environment and their implications for ecosystems: A review. Sci. Total. Environ..

[3] Dikshit R., Tallapragada P. (2018). Comparative Study of Natural and Artificial Flavoring Agents and Dyes. Natural and Artificial Flavoring Agents and Food Dyes.

[4] Sutthiwong N., Fouillaud M., Valla A., Caro Y., Dufossé L. (2014). Bacteria belonging to the extremely versatile genus Arthrobacter as novel source of natural pigments with extended hue range. Food Res. Int..

[5] Usman H.M., Abdulkadir N., Gani M., Maiturare H.M. (2017). Bacterial pigments and its significance. MOJ Bioequiv Availab..

[6] (2021). AMR. https://www.alliedmarketresearch.com/carotenoids-market.

[7] Mussagy C.U., Khan S., Kot A.M. (2021). Current developments on the application of microbial carotenoids as an alternative to synthetic pigments. Crit. Rev. Food Sci. Nutr..

[8] Ram S., Mitra M., Shah F., Tirkey S.R., Mishra S. (2020). Bacteria as an alternate biofactory for carotenoid production: A review of its applications, opportunities and challenges. J. Funct. Food.

[9] Usmani Z., Sharma M., Sudheer S., Gupta V.K., Bhat R. (2020). Engineered Microbes for Pigment Production Using Waste Biomass. Curr. Genom..

[10] Jaradat A., Al-Akhras MA H., Makhadmeh G., Aljarrah K., Al-Omari A., Ababneh Z., Alshorman M. (2011). Artificial semi-rigid sensitized with natural pigments: Effect of photon radiations. J. Pharm. BioAllied Sci..

[11] Mordini E., Ashton H., Mordini E., Tzovaras D. (2012). The Transparent Body: Medical Information, Physical Privacy and Respect for Body Integrity. Second Generation Biometrics: The Ethical, Legal and Social Context. The International Library of Ethics, Law and Technology.

[12] Fingerman M. (1965). Chromatophores. Physiol. Rev..

[13] Rudh A., Qvarnström A. (2013). Adaptive colouration in amphibians. Semin. Cell Dev. Biol..

[14] Price A.C., Weadick C.J., Shim J., Rodd F.H. (2008). Pigments, Patterns, and Fish Behavior. Zebrafish.

[15] Villanueva J., Grimalt J.O., de Wit R., Keely B.J., Maxwell J.R. (1194). Chlorophyll and carotenoid pigments in solar saltern microbial mats. Geochim. Cosmochim. Acta.

[16] Rajagopal L., Sundari S.C., Balasubramanian D., Sonti R. (1997). The bacterial pigment xanthomonadin offers protection against photodamage. FEBS Lett..

[17] Sen T., Barrow C.J., Deshmukh S.K. (2019). Microbial Pigments in the Food Industry—Challenges and the Way Forward. Front. Nutr..

[18] Ambati R.R., Siew Moi P., Ravi S., Aswathanarayana R.G. (2014). Astaxanthin: Sources, extraction, stability, biological activities and its commercial applications—A review. Marine Drugs.

[19] Galasso C., Corinaldesi C., Sansone C. (2017). Carotenoids from Marine Organisms: Biological Functions and Industrial Applications. Antioxidants.

[20] Cappelletti M., Presentato A., Piacenza E., Firrincieli A., Turner R.J., Zannoni D. (2020). Biotechnology of Rhodococcus for the production of valuable compounds. Appl. Microbiol. Biotechnol..

[21] Alshamaa D.S., Issam M.M. (2017). The Role of Extracted Carotenoid from Staphylococci as Antioxidant and Antibacterial. Rafidain J. Sci..

[22] Tian B., Sun Z., Shen S., Wang H., Jiao J., Wang L., Hu Y. (2009). Effects of carotenoids from *Deinococcus radiodurans* on protein oxidation. Lett. Appl. Microbiol..

[23] Williamson N.R. (2005). Biosynthesis of the red antibiotic, prodigiosin, in Serratia: Identification of a novel 2-methyl-3-n-amyl-pyrrole (MAP) assembly pathway, definition of the terminal condensing enzyme, and implications for undecylprodigiosin biosynthesis in Streptomyces. Mol. Microbiol..

[24] Stankovic N., Senerovic L., Ilic-Tomic T., Vasiljevic B., Nikodinovic-Runic J. (2014). Properties and applications of undecylprodigiosin and other bacterial prodigiosins. Appl. Microbiol. Biotechnol..

[25] Song Y., Liu G., Li J., Huang H., Zhang X., Zhang H., Ju J. (2015). Cytotoxic and Antibacterial Angucycline- and Prodigiosin- Analogues from the Deep-Sea Derived Streptomyces sp. SCSIO 11594. Mar. Drugs.

[26] Lazaro J.E., Nitcheu J., Predicala R.Z., Mangalindan G.C., Nesslany F., Marzin D., Diquet B. (2002). Heptyl prodigiosin, a bacterial metabolite, is antimalarial in vivo and non-mutagenic in vitro. J. Nat. Toxins.

[27] Baront S.S., Rowe J.J. (1981). Antibiotic Action of Pyocyanin. Antimicrob. Agents Chemother..

[28] Justo G.Z., Durán N., Ferreira C.V., Melon P.S., Cordi L., Martins D. (2007). Violacein: Properties and biological activities. Biotechnol. Appl. Biochem..

[29] Andrighetti-Fröhner C.R., Antonio R.V., Creczynski-Pasa T.B., Barardi C.R.M., O Simões C.M. (2003). Cytotoxicity and potential antiviral evaluation of violacein produced by Chromobacterium violaceum. Memórias Inst. Oswaldo Cruz.

[30] Lu Y., Wang L., Xue Y., Zhang C., Xing X.-H., Lou K., Zhang Z., Li Y., Zhang G., Bi J. (2009). Production of violet pigment by a newly isolated psychrotrophic bacterium from a glacier in Xinjiang, China. Biochem. Eng. J..

[31] Woodhams D.C., LaBumbard B.C., Barnhart K.L., Becker M.H., Bletz M.C., Escobar L.A., Flechas S.V., Forman M.E., Iannetta A.A., Joyce M.D. (2017). Prodigiosin, Violacein, and Volatile Organic Compounds Produced by Widespread Cutaneous Bacteria of Amphibians Can Inhibit Two Batrachochytrium Fungal Pathogens. Microb. Ecol..

[32] Rao M.P.N., Xiao M., Li W.-J. (2017). Fungal and Bacterial Pigments: Secondary Metabolites with Wide Applications. Front. Microbiol..

[33] Dharmaraj S., Ashokkumar B., Dhevendaran K. (2009). Food-grade pigments from *Streptomyces* sp. isolated from the marine sponge Callyspongia diffusa. Food Res. Int..

[34] Venil C.K., Dufossé L., Renuka Devi P. (2020). Bacterial Pigments: Sustainable Compounds with Market Potential for Pharma and Food Industry. Front. Sustain. Food Syst..

[35] Hathaway J.J.M., Garcia M.G., Balasch M.M., Spilde M.N., Stone F.D., Dapkevicius M.D.L.N.E., Northup D.E. (2014). Comparison of Bacterial Diversity in Azorean and Hawai’ian Lava Cave Microbial Mats. Geomicrobiol. J..

[36] Kristjánsson J.K., Hjörleifsdóttir S., Marteinsson V.T., Alfredsson G.A. (1994). Thermus scotoductus, sp. nov., a Pigment-Producing Thermophilic Bacterium from Hot Tap Water in Iceland and Including Thermus sp. X-1. Syst. Appl. Microbiol..

[37] Hermansson M., Jones G.W., Kjelleberg S. (1987). Frequency of antibiotic and heavy metal resistance, pigmentation, and plasmids in bacteria of the marine airwater interface. Appl. Environ. Microbiol..

[38] Sabbagh P., Namvar A.E. (2017). The eminence status of bacterial pigments under different aspects. Microbiol. Medica.

[39] Zhao J., Li Q., Sun T., Zhu X., Xu H., Tang J., Zhang X., Ma Y. (2013). Engineering central metabolic modules of *Escherichia coli* for improving β-carotene production. Metab. Eng..

[40] El-Naggar N.E.-A., El-Ewasy S.M. (2017). Bioproduction, characterization, anticancer and antioxidant activities of extracellular melanin pigment produced by newly isolated microbial cell factories Streptomyces glaucescens NEAE-H. Sci. Rep..

[41] Dorina S., Judith S., Bjoern W., Julia S., Andrea S., Sarah D.N., Roland U. (2020). A new strategy for combined isolation of EPS and pigments from cyanobacteria. J. Appl. Phycol..

[42] Shetty M.R.D.R. (2018). Screening and Extraction of Microbial Pigment from Organism Isolated from Marine Water. Int. J. Sci. Res. (IJSR).

[43] Yang J., Guo L. (2014). Biosynthesis of β-carotene in engineered E. coli using the MEP and MVA pathways. Microb. Cell Factories.

[44] Park H., Park S., Yang Y.-H., Choi K.-Y. (2021). Microbial synthesis of violacein pigment and its potential applications. Crit. Rev. Biotechnol..

[45] Di L.I., Yang L.I., Jiao-Yang X.U., Qing-Yan L.I., Jin-Lei T.A.N.G., Shi-Ru J.I.A., Chang-Hao B.I., Zhu-Bo D.A.I., Xin-Na Z.H.U., Zhang X.L. (2020). Engineering CrtW and CrtZ for improving biosynthesis of astaxanthin in Escherichia coli. Chin. J. Nat. Med..

[46] Nishizaki T., Tsuge K., Itaya M., Doi N., Yanagawa H. (2007). Metabolic engineering of carotenoid biosynthesis in Escherichia coli by ordered gene assembly in Bacillus subtilis. Appl. Environ. Microbiol..

[47] Pelz A., Wieland K.P., Putzbach K., Hentschel P., Albert K., Götz F. (2005). Structure and Biosynthesis of Staphyloxanthin from Staphylococcus aureus. J. Biol. Chem..

[48] Hannibal L., Lorquin J., D’Ortoli N.A., Garcia N., Chaintreuil C., Masson-Boivin C., Dreyfus B., Giraud E. (2000). Isolation and Characterization of Canthaxanthin Biosynthesis Genes from the Photosynthetic Bacterium *Bradyrhizobium* sp. Strain ORS278. J. Bacteriol..

[49] Blankenfeldt W., Parsons J.F. (2014). The structural biology of phenazine biosynthesis. Curr. Opin. Struct. Biol..

[50] Henke N.A., Wiebe D., Pérez-García F., Peters-Wendisch P., Wendisch V.F. (2018). Co-production of cell-bound and secreted value-added compounds: Simultaneous production of carotenoids and amino acids by Corynebacterium glutamicum. Bioresour. Technol..

[51] Li X.-R., Tian G.-Q., Shen H.-J., Liu J.-Z. (2015). Metabolic engineering of *Escherichia coli* to produce zeaxanthin. J. Ind. Microbiol. Biotechnol..

[52] Su A., Chi S., Li Y., Tan S., Qiang S., Chen Z., Meng Y. (2018). Metabolic redesign of *Rhodobacter sphaeroides* for lycopene production. J. Agric. Food Chem..

[53] Taniguchi H., Henke N.A., Heider S.A., Wendisch V.F. (2017). Overexpression of the primary sigma factor gene sigA improved carotenoid production by Corynebacterium glutamicum: Application to production of β-carotene and the non-native linear C50 carotenoid bisanhydrobacterioruberin. Metab. Eng. Commun..

[54] della-Cioppa G., Garger S.J., Sverlow G.G., Turpen T.H., Grill L.K. (1990). Melanin production in Escherichia coli from a cloned tyrosinase gene. Biotechnology.

[55] Yoon S.-H., Kim J.-E., Lee S.-H., Park H.-M., Choi M.-S., Kim J.-Y., Lee S.-H., Shin Y.-C., Keasling J., Kim S.-W. (2007). Engineering the lycopene synthetic pathway in E. coli by comparison of the carotenoid genes of Pantoea agglomerans and Pantoea ananatis. Appl. Microbiol. Biotechnol..

[56] Pan M.H., Ho C.T. (2008). Chemopreventive effects of natural dietary compounds on cancer development. Chem. Soc. Rev..

[57] Siegel R.L., Miller K.D., Jemal A. (2019). Cancer statistics. CA A Cancer J. Clin..

[58] Bagchi D., Swaroop A., Preuss H.G., Bagchi M. (2014). Free radical scavenging, antioxidant and cancer chemoprevention by grape seed proanthocyanidin: An overview. Mutat. Res.-Fundam. Mol. Mech. Mutagen..

[59] Fernandes C.J., Doddavarapu B., Harry A., Dilip S.P.S., Ravi L. (2021). Isolation and Identification of Pigment Producing Actinomycete Saccharomonospora azurea SJCJABS01. Biomed. Pharmacol. J..

[60] Abraham J., Chauhan R. (2018). Profiling of red pigment produced by Streptomyces sp. JAR6 and its bioactivity. 3 Biotech.

[61] Selvameenal L., Radhakrishnan M., Balagurunathan R. (2009). Antibiotic pigment from desert soil actinomycetes; biological activity, purification and chemical screening. Indian J. Pharm. Sci..

[62] Shaaban M.T., El-Sabbagh S.M.M., Alam A. (2013). Studies on an actinomycete producing a melanin pigment inhibiting aflatoxin B1 production by Aspergillus flavus. Life Sci. J..

[63] Chang Y., Xing L., Sun C., Liang S., Liu T., Zhang X., Zhu T., Pfeifer B.A., Che Q., Zhang G. (2020). Monacycliones G–K and *ent*-Gephyromycin A, Angucycline Derivatives from the Marine-Derived *Streptomyces* sp. HDN15129. J. Nat. Prod..

[64] Ramirez-Rodriguez L., Stepanian-Martinez B., Morales-Gonzalez M., Diaz L. (2018). Optimization of the Cytotoxic Activity of Three *Streptomyces* Strains Isolated from Guaviare River Sediments (Colombia, South America). BioMed Res. Int..

[65] Prashanthi K., Suryan S., Varalakshmi K.N. (2015). In vitro anticancer property of yellow pigment from Streptomyces griseoaurantiacus JUACT 01. Braz. Arch. Biol. Technol..

[66] Hegazy G.E., Abu-Serie M.M., Abo-Elela G.M., Ghozlan H., Sabry S.A., Soliman N.A., Abdel-Fattah Y.R. (2020). In vitro dual (anticancer and antiviral) activity of the carotenoids produced by haloalkaliphilic archaeon Natrialba sp. M6. Sci. Rep..

[67] Moayedi A., Nowroozi J., Sepahi A.A. (2018). Cytotoxic effect of pyocyanin on human pancreatic cancer cell line (Panc-1). Iran J. Basic Med. Sci..

[68] Vipin C., Ashwini P., Kavya A., Rekha P. (2017). Overproduction of Pyocyanin in *Pseudomonas aeruginosa* by Supplementation of Pathway Precursor Shikimic acid and Evaluation of its Activity. Res. J. Pharm. Technol..

[69] Stankovic N., Radulovic V., Petkovic M., Vuckovic I., Jadranin M., Vasiljevic B., Nikodinovic-Runic J. (2012). Streptomyces sp. JS520 produces exceptionally high quantities of undecylprodigiosin with antibacterial, antioxidative, and UV-protective properties. Appl. Microbiol. Biotechnol..

[70] Charkoudian L.K., Fitzgerald J.T., Khosla C., Champlin A. (2010). In Living Color: Bacterial Pigments as an Untapped Resource in the Classroom and Beyond. PLoS Biol..

[71] Teruel M.A., Gontier E., Bienaime C., Saucedo J.N., Barbotin J.N. (1997). Response surface analysis of chlortetracycline and tetracycline production with K-carraimmobilizedbilized Streptomyces aureofaciens. Enzym. Microb. Technol..

[72] Rutherford S.T., Bassler B.L. (2012). Bacterial Quorum Sensing: Its Role in Virulence and Possibilities for Its Control. Cold Spring Harb. Perspect. Med..

[73] Naclerio G., Ricca E., Sacco M., De Felice M. (1993). Antimicrobial activity of a newly identified bacteriocin of Bacillus cereus. Appl. Environ. Microbiol..

[74] Baron S.S., Terranova G., Rowe J.J. (1989). Molecular mechanism of the antimicrobial action of pyocyanin. Curr. Microbiol..

[75] Raji El Feghali P.A., Nawas T. (2018). Pyocyanin: A powerful inhibitor of bacterial growth and biofilm formation. Madridge J. Case Rep. Stud..

[76] Akaike T. (2001). Role of free radicals in viral pathogenesis and mutation. Rev. Med. Virol..

[77] Brunelle M.-N., Jacquard A.-C., Pichoud C., Durantel D., Carrouée-Durantel S., Villeneuve J.-P., Trépo C., Zoulim F. (2005). Susceptibility to antivirals of a human HBV strain with mutations conferring resistance to both lamivudine and adefovir. Hepatology.

[78] Zhou W., Zeng C., Liu R., Chen J., Li R., Wang X., Bai W., Liu X., Xiang T., Zhang L. (2015). Antiviral activity and specific modes of action of bacterial prodigiosin against Bombyx mori nucleopolyhedrovirus in vitro. Appl. Microbiol. Biotechnol..

[79] Suryawanshi R.K., Koujah L., Patil C.D., Ames J.M., Agelidis A., Yadavalli T., Patil S.V., Shukla D. (2020). Bacterial Pigment Prodigiosin Demonstrates a Unique Antiherpesvirus Activity That Is Mediated through Inhibition of Prosurvival Signal Transducers. J. Virol..

[80] Durán M., Faljoni-Alario A., Durán N. (2010). Chromobacterium violaceum and its important metabolites—Review. Folia Microbiol..

[81] Lionakis M.S. (2012). Genetic Susceptibility to Fungal Infections in Humans. Curr. Fungal Infect. Rep..

[82] Thompson G.R., Cadena J., Patterson T.F. (2009). Overview of Antifungal Agents. Clin. Chest Med..

[83] Jimtha C.J., Jishma P., Sreelekha S., Chithra S., Radhakrishnan E. (2017). Antifungal properties of prodigiosin producing rhizospheric *Serratia* sp.. Rhizosphere.

[84] Dawoud T.M., Alharbi N.S., Theruvinthalakal A.M., Thekkangil A., Kadaikunnan S., Khaled J.M., Almanaa T.N., Sankar K., Innasimuthu G.M., Alanzi K.F. (2019). Characterization and antifungal activity of the yellow pigment produced by a Bacillus sp. DBS4 isolated from the lichen Dirinaria agealita. Saudi J. Biol. Sci..

[85] Wagh P., Mane R. (2017). Identification and characterization of extracellular red pigment producing Neisseria spp. isolated from soil sample. Int. J. Innov. Knowl. Concept.

[86] Houshaymi B., Awada R., Kedees M., Soayfane Z. (2019). Pyocyanin, a Metabolite of Pseudomonas Aeruginosa, Exhibits Antifungal Drug Activity Through Inhibition of a Pleiotropic Drug Resistance Subfamily FgABC3. Drug Res..

[87] Sudhakar T., Karpagam S. Antifungal efficacy of pyocyanin produced from bioindicators of nosocomial hazards. Proceedings of the International Conference on Green technology and environmental Conservation (GTEC-2011).

[88] Kerr J.R., Taylor G.W., Rutman A., Hoiby N., Cole P.J., Wilson R. (1999). Pseudomonas aeruginosa pyocyanin and 1-hydroxyphenazine inhibit fungal growth. J. Clin. Pathol..

[89] Mahmoud S.Y., Ziedan E.S.H., Farrag E.S., Kalafalla R.S., Ali A.M. (2016). Antifungal activity of pyocyanin produced by Pseudomonas aeruginosa against Fusarium oxysporum Schlech phytopathogenic fungi. Int. J. PharmTech Res..

[90] Bisht G., Srivastava S., Kulshreshtha R., Sourirajan A., Baumler D.J., Dev K. (2020). Applications of red pigments from psychrophilic Rhodonellum psychrophilum GL8 in health, food and antimicrobial finishes on textiles. Process. Biochem..

[91] Mitra A.K., Mawson A.R. (2020). Neglected Tropical Diseases: Epidemiology and Global Burden. Trop. Med. Infect. Dis..

[92] Silva T.R., Duarte A.W.F., Passarini M.R.Z., Ruiz A.L.T.G., Franco C.H., Moraes C.B., de Melo I.S., Rodrigues R.A., Fantinatti-Garboggini F., Oliveira V.M. (2018). Bacteria from Antarctic environments: Diversity and detection of antimicrobial, antiproliferative, and antiparasitic activities. Polar Biol..

[93] Bilsland E., Tavella T.A., Krogh R., Stokes J.E., Roberts A., Ajioka J., Spring D.R., Andricopulo A.D., Costa F.T.M., Oliver S.G. (2018). Antiplasmodial and trypanocidal activity of violacein and deoxyviolacein produced from synthetic operons. BMC Biotechnol..

[94] Rahul S., Chandrashekhar P., Hemant B. (2014). Natural Product Research: Formerly Natural Product Letters Nematicidal activity of microbial pigment from Serratia marcescens. Nat. Prod. Res..

[95] Gutierrez-dalmau A., Campistol J.M. (2007). Immunosuppressive Therapy and Malignancy in Organ A Systematic Review. Drugs.

[96] Kawauchiab K., Shibutaniab K., Yagisawaa H., Kamataa H., Nakatsuji S., Anzaic H., Yokoyamab Y., Ikegamib Y., Moriyamad Y., Hirata H. (1997). A Possible Immunosuppressant, Cycloprodigiosin Hydrochloride, Obtained from Pseudoalteromonas denitrificans. Biochem. Biophys. Res. Commun..

[97] Nakashima T., Kurachi M., Kato Y., Yamaguchi K., Oda T. (2005). Characterization of Bacterium Isolated from the Sediment at Coastal Area of Omura Bay in Japan and Several Biological Activities of Pigment Produced by This Isolate. Microbiol. Immunol..

[98] Pandey R., Chander R., Sainis K. (2003). A novel prodigiosin-like immunosuppressant from an alkalophilic *Micrococcus* sp.. Int. Immunopharmacol..

[99] Burrows J.A. (2009). Palette of Our Palates: A Brief History of Food Coloring and Its Regulation. Compr. Rev. Food Sci. Food Saf..

[100] Downham A., Collins P. (2000). Colouring our foods in the last and next millennium. Int. J. Food Sci. Technol..

[101] Manikprabhu D., Lingappa K. (2013). γ Actinorhodin a natural and attorney source for synthetic dye to detect acid production of fungi. Saudi J. Biol. Sci..

[102] Galaffu N., Bortlik K., Michel M. (2015). An industry perspective on natural food colour stability. Colour Additives for Foods and Beverages.

[103] Wrolstad R.E., Culver C.A. (2012). Alternatives to Those Artificial FD&C Food Colorants. Annu. Rev. Food Sci. Technol..

[104] Özkan G., Bilek S.E. (2014). Microencapsulation of Natural Food Colourants. Int. J. Nutr. Food Sci..

[105] Silva P.I., Stringheta P.C., Teófilo R.F., de Oliveira I.R.N. (2013). Parameter optimization for spray-drying microencapsulation of jaboticaba (Myrciaria jaboticaba) peel extracts using simultaneous analysis of responses. J. Food Eng..

[106] Rocha G.A., Fávaro-Trindade C.S., Grosso C.R.F. (2012). Microencapsulation of lycopene by spray drying: Characterization, stability and application of microcapsules. Food Bioprod. Process..

[107] Heer K., Sharma S. (2017). Microbial pigments as a natural colour—A review. Int. J. Pharm. Sci. Res..

[108] Yokoyama A. (1995). Composition and presumed biosynthetic pathway of carotenoids in the astaxanthin-producing bacterium. FEMS Microbiol. Lett..

[109] Surai P.F. (2012). The antioxidant properties of canthaxanthin and its potential effects in the poultry eggs and on embryonic development of the chick. Part 2. World’s Poult. Sci. J..

[110] Wagner-Döbler I., Beil W., Lang S., Meiners M., Laatsch H. (2002). Integrated approach to explore the potential of marine microorganisms for the production of bioactive metabolites. Tools and Applications of Biochemical Engineering Science.

[111] Guillerme J.B., Couteau C., Coiffard L. (2017). Applications for marine resources in cosmetics. Cosmetics.

[112] Tian B., Hua Y. (2010). Carotenoid biosynthesis in extremophilic Deinococcus-Thermus bacteria. Trends Microbiol..

[113] Wan M., Hou D., Li Y., Fan J., Huang J., Liang S., Li S. (2014). The effective photoinduction of Haematococcus pluvialis for accumulating astaxanthin with attached cultivation. Bioresour. Technol..

[114] Shindo K., Misawa N. (2014). New and rare carotenoids isolated from marine bacteria and their antioxidant activities. Mar. Drugs.

[115] Rittié L., Fisher G.J. (2002). UV-light-induced signal cascades and skin aging. Ageing Res. Rev..

[116] Sorrels C.M., Proteau P.J., Gerwick W.H. (2009). Organization, evolution, and expression analysis of the biosynthetic gene cluster for scytonemin, a cyanobacterial UV-absorbing pigment. Appl. Environ. Microbiol..

[117] Rastogi R.P., Sinha R.P. (2013). Incharoensakdi, Acharacterizationterization, UV-induction and photoprotective function of sunscreen pigment, scytonemin from Rivularia sp. HKAR-4. Chemosphere.

[118] Meot-Duros L., Cérantola S., Talarmin H., Le Meur C., LE Floch G., Magné C. (2010). New antibacterial and cytotoxic activities of falcarindiol isolated in Crithmum maritimum L. leaf extract. Food Chem. Toxicol..

[119] Alihosseini F., Ju K.S., Lango J., Hammock B.D., Sun G. (2008). Antibacterial colorants: Characterization of prodiginines and their applications on textile materials. Biotechnol. Prog..

[120] Venil C.K., Dufossé L., Velmurugan P., Malathi M., Lakshmanaperumalsamy P. (2021). Extraction and application of pigment from Serratia marcescens SB08, an insect enteric gut bacterium, for textile dyeing. Textiles.

[121] Sajjad W., Ahmad S., Aziz I., Azam S.S., Hasan F., Shah A.A. (2018). Antiproliferative, antioxidant and binding mechanism analysis of prodigiosin from newly isolated radio-resistant Streptomyces sp. Strain WMA-LM31. Mol. Biol. Rep..

[122] Montaner B., Prez-Toms R. (2003). The prodigiosins: A new family of anticancer drugs. Curr. Cancer Drug Targets.

[123] Cao W., Chen W., Sun S., Guo P., Song J., Tian C. (2007). Investigating the antioxidant mechanism of violacein by density functional theory method. J. Mol. Struct. Theochem..

[124] Suryawanshi R.K., Patil C.D., Borase H.P., Narkhede C.P., Stevenson A., Hallsworth J.E., Patil S.V. (2015). Towards an understanding of bacterial metabolites prodigiosin and violacein and their potential for use in commercial sunscreens. Int. J. Cosmet. Sci..

[125] Britton G. (1995). Structure and properties of carotenoids in relation to function. FASEB J..

